# Elevated c-Src is linked to altered cell–matrix adhesion rather than proliferation in KM12C human colorectal cancer cells

**DOI:** 10.1038/sj.bjc.6600594

**Published:** 2002-11-04

**Authors:** R J Jones, E Avizienyte, A W Wyke, D W Owens, V G Brunton, M C Frame

**Affiliations:** Beatson Institute for Cancer Research, Cancer Research UK Beatson Laboratories, Garscube Estate, Switchback Road, Bearsden, Glasgow G61 1BD, UK; Institute of Biological and Life Sciences, University of Glasgow, Glasgow G12 8QQ, UK; School of Life Sciences, University of Dundee, Dundee DD1 5EH, UK

**Keywords:** Src, kinase, colon cancer, growth, adhesion

## Abstract

Elevated expression and/or activity of c-Src, the prototype of the Src family of protein tyrosine kinases, is associated with the development of human colon cancer. However, despite the known pleiotropic effects of these kinases in promoting (a) cell growth downstream of growth factor receptors, and (b) the dynamic regulation of integrin adhesions in fibroblast model systems, their precise role in epithelial cancer cells is unknown. Here we addressed whether elevated expression and activity of cellular Src alters cell proliferation and/or cell–matrix adhesion in cancer cells from the Fidler model of colorectal metastasis. Although elevated Src correlates with ability to metastasise to the liver after intrasplenic injection, we found that this was not linked to enhanced growth, either *in vitro* or *in vivo* as sub-cutaneous tumours. However, elevated Src was associated with enhanced attachment to extracellular matrix. In addition, adhesion to fibronectin, was suppressed by agents that inhibited Src activity, while enforced elevation of Src in non-metastatic cells was sufficient to stimulate adhesion to fibronectin and enhanced assembly of adhesion complexes, without influencing cell growth. Thus, we conclude that one role of elevated Src in human colon cancer cells is to modulate integrin-dependent cell–matrix attachment and formation of adhesion structures, which may, in turn, influence cell motility and integrin-dependent cellular responses.

*British Journal of Cancer* (2002) **87**, 1128–1135. doi:10.1038/sj.bjc.6600594 www.bjcancer.com

© 2002 Cancer Research UK

## 

Although metastatic spread is the major cause of death in cancer patients, the molecular mechanisms that control key steps in the dissemination process remain elusive. Although analysis of cancer cells derived from metastases is not, by itself, able to decipher the metastatic process, understanding how individual oncoproteins or tumour suppressor proteins contribute to the fundamental biological changes that accompany metastasis, can provide insight into the control of events that promote metastasis. In this regard, cellular models, such as that provided by the Fidler model of colorectal metastasis (Morikawa *et al*, 1988), provide tractable reductionist systems. Here we have used cancer cells from the Fidler model to specifically address the behavioural changes associated with elevated expression and activity of the c-Src tyrosine kinase that frequently occurs in metastatic colorectal cells.

A number of studies have demonstrated elevated cellular activity of c-Src, the prototypic member of the Src family of protein tyrosine kinases, during colorectal tumour progression, with an increasing trend from adenomas through to malignant carcinomas and metastases (Bolen *et al*, 1987; Cartwright *et al*, 1989, 1990, 1994; Talamonti *et al*, 1993; Termuhlen *et al*, 1993). It was also recently reported that an activating mutation of c-Src, at tyrosine 527 in the carboxy-terminal regulatory region, occurred in a sub-set of advanced, metastatic colon cancers (Irby *et al*, 1999). However, mutational activation of c-Src has not been found in other similar studies (Daigo *et al*, 1999; Wang *et al*, 2000a; Laghi *et al*, 2001), and so there remains uncertainty about the mode of c-Src elevation in colorectal cancer cells. Here we address how c-Src contributes to the behaviour of colorectal cancer cells, specifically asking whether elevated cellular c-Src activity alters colon cancer cell growth and/or adhesion, two properties that are influenced by Src in model cell systems and that are frequently altered during colorectal cancer progression.

Studies over many years in fibroblast model systems have implicated the Src family kinases (SFKs) in growth factor mediated mitogenic responses (Twamley *et al*, 1993; Roche *et al*, 1995). Specifically, c-Src is required for platelet-derived growth factor (PDGF)-induced mitogenesis (Roche *et al*, 1995; Twamley *et al*, 1993), most likely via its induction of a Stat3/c-Myc pathway (Barone and Courtneidge, 1995; Broome and Hunter, 1996; Wang *et al*, 2000b; Bowman *et al*, 2001). Also, v-Src can induce mitogenesis in serum-deprived fibroblasts (Welham *et al*, 1990) as well as promote the G1/S phase transition in growing transformed cells (Johnson *et al*, 1998).

There is considerable evidence that c-Src can also regulate adhesion dynamics by both kinase-dependent and kinase-independent mechanisms. Exogenous expression of c-Src can stimulate cell–matrix adhesion of c-Src −/− fibroblasts by a mechanism that is independent of its catalytic activity (Kaplan *et al*, 1995), while kinase-independent functions of v-Src are required for its incorporation into newly assembling integrin adhesions (Fincham and Frame, 1998; Fincham *et al*, 2000). Recent work also showed that cells derived from triple Src/Fyn/Yes null embryos display impaired motility (Klinghoffer *et al*, 1999), while the dominant negative effect of kinase-deficient v-Src suppresses adhesion turnover and cell migration (Fincham and Frame, 1998). These activities of SFKs are not mutually exclusive and SFK activity at the cell membrane may co-ordinate adhesion dependent growth responses. Here we have addressed whether elevated c-Src in metastatic cells correlates with enhanced growth or enhanced cell–matrix adhesion, and whether expression of exogenous active c-Src in the non-metastatic cells is sufficient to induce either of these.

## MATERIALS AND METHODS

### Cell culture and cell lines

Cell lines KM12 C, KM12 SM and KM12 L4A were a kind gift from Professor I Fidler (MD Anderson, TX, USA) and the RGC2 cells were a gift from Professor C Paraskeva (Bristol, UK). KM12 cells were cultured in Eagles MEM with Earle's salts supplemented with MEM vitamins (×2), non-essential amino acids, L-glutamine (2 mM) and sodium pyruvate (1 mM) (all from Life Technologies, Paisley, UK) in the presence of 10% foetal bovine serum (Autogen Bioclear, Wiltshire, UK). RGC2 cells were cultured in Dulbecco's modified Eagle's medium with hydrochloric acid (4 mM), sodium bicarbonate (0.075%), glutamine (2 mM), (Life Technologies) supplemented with 20% foetal bovine serum (Autogen Bioclear), insulin (10 μg ml^−1^, Roche Diagnostics Ltd, Lewes, UK) and hydrocortisone (1 μg ml^−1^, Sigma Chemical Company). All cells were routinely maintained in a humidified incubator at 37°C with 5% CO_2_ and sub-cultured before reaching confluence.

KM12C cells were transfected using the Lipofectamine Plus reagent (Life Technologies) and stable cell lines derived after selection of single cell clones. The cDNA expression vector, pUSE (Upstate Biotechnology) was used to derive cell lines expressing the vector alone and active c-Src (Y527F).

### Anchorage-independent growth

Culture medium containing 20% serum, 50 U ml^−1^ penicillin, 250 μg ml^−1^ streptomycin and 0.6% Agar was prepared. Four ml of agar/medium was placed into 60 mm tissue culture dishes and allowed to solidify (base agar). Cells were re-suspended at 5×10^2^ cells ml^−1^ in medium containing 0.3% agar and 2 ml layered on top of the base agar. Colony growth was recorded after 7 days.

## Tumourigenicity assay

Cells for injection were resuspended in Hanks' buffered saline solution (HBSS) at 37°C prior to subcutaneous injection of 2×10^6^ cells in 100 μl of HBSS into the right flank of 4–6-week-old female nude mice (Charles Rivers, Harlan, UK). Bidimensional tumour measurements were made every 5 days using callipers and the average of these measurements used as an estimate of the diameter of a sphere in order to calculate tumour volume.

### Immunoblotting and immunoprecipitation

Cell extracts were prepared in RIPA buffer (50 mM Tris, 150 mM NaCl, 1% NP40, 0.5% deoxycholate, 0.1% SDS, pH 7.4) from sub-confluent cell cultures and clarified by centrifugation at 4°C. Total protein was measured using microBCA reagent (Pierce, Rockford, IL, USA). Immunoprecipitates of c-Src phosphorylated on tyrosine-416 were prepared by incubating 1.5 mg of cell lysate with 2 μg of polyclonal phosphotyrosine-416-c-Src-specific antibody (Biosource International, Camarillo, CA, USA) overnight. Paxillin immunoprecipitates were prepared from 1 mg of protein with 4 μg anti-paxillin antibody (Transduction Laboratories, Lexington, KY, USA). Immune-complexes were collected using protein A sepharose (Sigma Chemical Company, Poole, UK) and washed extensively with lysis buffer. Proteins were resolved by 10% (c-Src) and 6% (phospho 416 Src) SDS–PAGE gels respectively, and transferred to nitrocellulose membranes prior to visualisation using anti-v-Src mAb (Ab-1) (0.1 μg ml^−1^, Oncogene Research Products, Cambridge, MA, USA). Paxillin immunoprecipitates were resolved by 7% SDS–PAGE gels prior to transfer and probed with anti-phosphotyrosine mAB PY20 (1 μg ml^−1^, Transduction Laboratories). Blots were subsequently stripped and reprobed with anti-paxillin antibody (25 ng ml^−1^). For immunoblotting, 50 μg cellular protein were resolved as above.

### Immune-complex kinase assays

Anti-c-Src from 200 μg cell lysate was immunoprecipitated with 2 μg EC10, collected on protein A-sepharose beads, washed extensively and assayed for protein kinase activity using 10 μg exogenous enolase as substrate. The kinase assay buffer was 20 mM PIPES pH 6.4, 20 mM MnCl_2_, 1 mM DTT, 100 μM sodium orthovanadate, with 10 μM ATP and 5 μCi ATP-[P^32^] (specific activity 3000 Cimmol^−1^). Incubation was for 10 min at 30°C, and the reaction was stopped by addition of SDS–PAGE sample buffer and boiling, prior to resolving proteins by SDS–PAGE. SU6656 was dissolved in DMSO to 10 mM stock, and diluted in cell culture medium. A similar amount of DMSO solvent was added to control cells.

### Microscopy and immunofluorescence

Glass coverslips were pre-coated with human fibronectin (Becton Dickinson, Bedford, MA, USA) or poly-L-lysine (Sigma) at 37°C for 90 min (10 μg ml^−1^ in phosphate buffered saline) and washed in PBS. Cells were plated for 60 min and fixed for 15 min in 3.7% formaldehyde at room temperature. After permeabilisation with 0.5% Triton X-100 for 10 min, cells were blocked with 10% foetal bovine serum before incubating overnight with anti-Src antibody N2-17 (1:750 dilution of ascites, a kind gift from T Hunter, Salk Institute, CA, USA) or anti-human vinculin (10 μg ml^−1^, Sigma). Antibody detection was with FITC-conjugated goat anti-mouse IgG (7.5 μg ml^−1^, Jackson Laboratories, Bar Harbor, MN, USA) for 45 min at room temperature. Cells were visualised using a confocal microscope (model MRC600; BioRad Labs, Hercules, CA, USA).

### Adhesion assay

96-well plastic plates (Falcon, Becton Dickinson, Oxnard, CA, USA) were coated with human fibronectin (Becton Dickinson), poly-L-lysine, vitronectin, laminin or type IV human collagen (all from Sigma). 0.1% bovine serum albumen (BSA) was added for 2 h at 37°C to block non-specific binding. Negative control wells were uncoated but blocked with BSA. Cells were suspended in complete medium and labelled with 90 μCi Chromium-51 (Amersham, Buckinghamshire, UK) at 37°C for 1 h with regular mixing. After washing four times in serum-free medium (0.5%), cells were resuspended in low-serum medium at 2.5×10^4^ ml^−1^and 50 μl^−1^ added to each well. The plates were incubated for 45 min at 37°C then the wells were gently washed twice with cold PBS. Individual wells were cut from the plate and the number of attached cells measured by gamma scintigraphy. A known number of cells was also counted in suspension in order to calibrate the activity per cell. Non-specific attachment was determined on the serum albumen-coated wells and this figure subtracted from the experimental values.

## RESULTS

### Elevated c-Src expression and cellular activity in metastatic cells from the Fidler model of colon cancer metastasis

**Figure 1:**
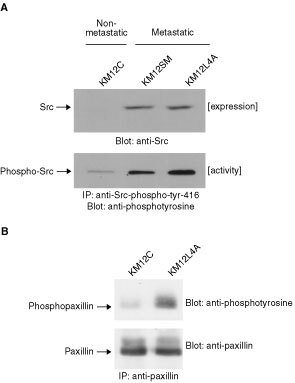
(**A**) Immunoblots showing expression (upper panel) and activity (lower panel) of c-Src in the poorly metastatic cell line, KM12C, and in their highly metastatic derivative cell lines, KM12SM and KM12L4A. Activity was assessed by reactivity with a phospho-specific antibody raised against the region of c-Src containing tyrosine-416, the presumed site of autophosphorylation. (**B**) Comparison of paxillin tyrosine phosphorylation in KM12C and KM12L4A cells. Paxillin was immunoprecipitated, blotted and probed with phosphotyrosine-specific antibodies (upper panel) or anti-paxillin (lower panel).

Most of the molecular studies on Src family kinases (SFKs) have been carried out in fibroblasts. Consequently, much less is known about SFK function in epithelial cells that are the targets of cancerous transformation in humans. We set out to address how elevated c-Src contributes to the behaviour of colon cancer cells, specifically examining cell growth and adhesion to extracellular matrix components. For these experiments we used cell lines from the Fidler model of colorectal metastasis (Morikawa *et al*, 1988). In this model, cells that have low intrinsic capacity to metastasise to the liver after intrasplenic injection (KM12C) were compared with derivative cell lines that have much greater metastatic capacity in this assay (KM12SM and KM12L4A). The generation and characterisation of these cell lines has been described (Morikawa *et al*, 1988). Importantly, we confirmed that the actual cells we used in our studies retained the characteristics of originally reported Fidler cell lines with respect to metastatic capacity (not shown), and the acquisition of metastatic potential correlated with elevated c-Src expression (Figure 1A

, upper panel). By using an antibody that reacts specifically with active c-Src when it is autophosphorylated at tyrosine-416, we showed that a parallel increase in c-Src activity occurred during metastatic conversion (Figure 1A, lower panel; similar finding reported in (Mao *et al*, 1997)). This indicated that the elevated c-Src protein in the metastatic cells was active. We also found that tyrosine phosphorylation of paxillin, a focal adhesion protein that is a substrate of c-Src in colon cancer cells (Rodina *et al*, 1999), was elevated in the metastatic cell lines (shown for KM12L4A in Figure 1B), implying that the additional c-Src protein was active at peripheral sites. Thus, metastatic potential of these colon cancer cells correlates with elevated c-Src expression and activity.

### Elevated c-Src is not linked to growth enhancement

**Figure 2:**
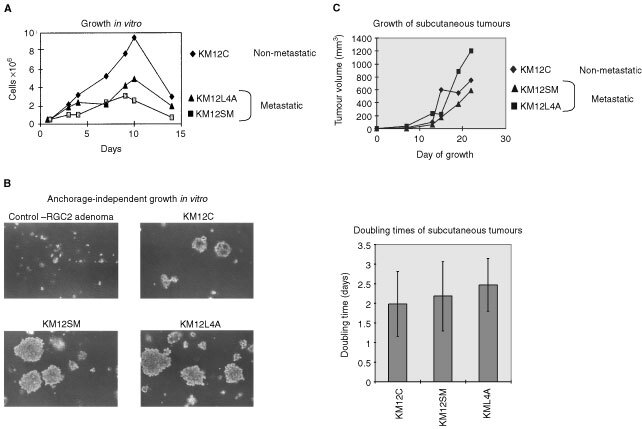
(**A**) *In vitro* growth of KM12C, KM12L4A and KM12SM cells (seeded at 1×10^5^ cells in 35 mm dishes) was monitored for 14 days. (**B**) The ability of KM12C, KM12SM and KM12L4A cells (seeded at 5×10^2^ cells per ml of medium containing 0.6% agar) to grow when deprived of anchorage was compared. As control, the colon adenoma cell line RGC2 that does not grow in semi-solid medium was used. Shown are representative areas on culture dishes. Over a number of experiments, there was no visible difference between the number, or size, of colonies formed by all three cell lines under anchorage-independent conditions. (**C**) Subcutaneous primary tumour growth of KM12C, KM12SM and KM12L4A cells was monitored by injecting 2×10^6^ cells into mice and measuring tumour dimensions at regular intervals. Tumour volumes (upper panel) and doubling times (lower panel) are shown. 4–6 mice were used in each experiment.

We examined KM12C cell growth *in vitro*, and compared with the metastatic counterparts (KM12L4A and KM12SM) (Figure 2A

). We did not observe enhanced growth of the metastatic variants that expressed elevated levels of c-Src; in fact, these cells increased in number less rapidly than the parental non-metastatic cells (Figure 2A). We also found both the non-metastatic and metastatic cells grew similarly under anchorage-independent conditions, one of the recognised *in vitro* hallmarks of malignant cells (Figure 2B). In addition, we found similar growth rate of tumours that arose after subcutaneous inoculation of nude mice with non-metastatic or metastatic cells (Figure 2C). Thus, elevated expression and activity of c-Src in the metastatic cells did not correlate with increased growth *in vitro* or *in vivo*.

### Elevated c-Src is associated with integrin adhesion assembly in metastatic cells

**Figure 3:**
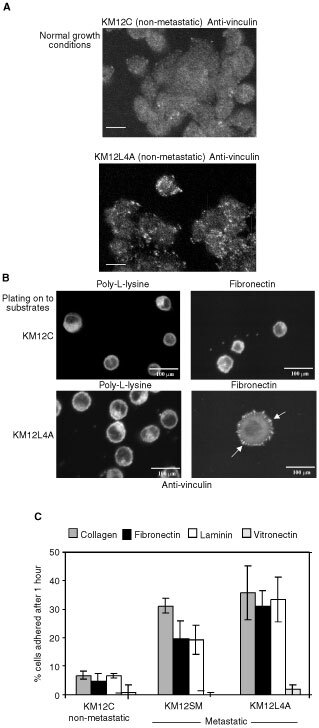
(**A**) Immunofluorescence staining (anti-vinculin) of KM12C and KM12L4A cells grown under normal culture conditions. Visualisation was by reaction of primary antibody with FITC-conjugated secondary antibody and confocal microscopy. Bars are 100 μm. (**B**) Anti-vinculin staining of KM12C and KM12L4A cells after plating suspended cells on to poly-L-lysine (left panels) or fibronectin (right panels) for 60 min. Bars are 100 μm. Similar images to those shown for KM12L4A cells were obtained for KM12SM cells. (**C**) The ability of KM12C, KM12SM and KM12L4A cells to attach to different matrix components was monitored using Chromium-51-labelled cells that were plated on to collagen, fibronectin, laminin or vitronectin for 45 min at 37°C. A known number of cells in suspension was also counted to calibrate the Chromium-51 counts per cell, allowing estimation of the number of attached cells. The means and standard errors of three replicates are presented.

As well as growth responses in fibroblasts (reviewed in Abram and Courtneidge, 2000), SFKs also influence cell adhesion in both fibroblasts (Fincham and Frame, 1998) and osteoclasts (Schwartzberg *et al*, 1997). We found that cultures of KM12L4A metastatic cells contained substantially more vinculin-containing peripheral adhesion structures than their KM12C non-metastatic counterparts under normal growth conditions (Figure 3A

). Furthermore, the KM12L4A metastatic cells formed peripheral adhesions more readily when plated on to fibronectin, as compared to their KM12C non-metastatic counterparts (Figure 3B, right panels). The formation of clearly discernable vinculin-containing structures was integrin dependent since these were not formed when the cells were plated on to poly-L-lysine (Figure 3B, left panels). In addition, the two metastatic variants, KM12SM and KM12L4A, displayed substantially greater adhesion to collagen, fibronectin and laminin than the KM12C non-metastatic counterparts (Figure 3C), while adhesion to vitronectin was minimal and indistinguishable in all three cell lines (Figure 3C). Taken together, these data indicate that elevated expression of c-Src in colon cancer cells from the Fidler model correlates with enhanced cell–matrix adhesion that is integrin-dependent. Integrin-dependence has now been more precisely defined by use of inhibitory antibodies to αv and β1 integrin subunits that specifically block adhesion of these colon cancer cells to fibronectin; cell surface expression of these integrins is not affected by over-expression of c-Src (not shown).

### Short-term treatment with an inhibitor of c-Src suppresses cell–matrix adhesion

**Figure 4:**
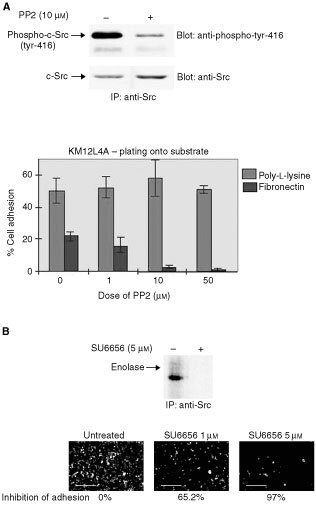
The effects of the Src inhibitory agents PP2 and SU6656 in KM12L4A cells. (**A**) PP2 suppresses Src autophosphorylation. c-Src phosphorylation on tyrosine-416 in the presence (+) and absence (−) of 10 μm PP2 for 60 min was determined by immunoprecipitating c-Src, blotting and probing with anti-phospho-tyrosine-416 (upper panels). c-Src expression was checked by probing blots with anti-Src (lower panels). The ability of PP2 (at concentrations ranging from 0 to 50 μm) to interfere with KM12L4A cell attachment to poly-L-lysine or to fibronectin was examined by Chromium-51 labelling. Suspended, labelled cells were treated with PP2 for 60 min prior to attaching for 60 min (lower panels). (**B**) SU6656 suppresses c-Src kinase activity. The ability of c-Src from cells grown in the presence and absence of SU6656 to phosphorylate enolase was determined by *in vitro* kinase assays (upper panels). The ability of SU6656 to suppress cell adhesion to fibronectin in 60 min was visualised microscopically and was quantitated as percentage inhibition of adhesion (lower panels). Bars are 125 μm.

To further probe c-Src's role in the behaviour of colon cancer cells from the Fidler model, we used a tyrosine kinase inhibitor (PP2) that is able to suppress c-Src autophosphorylation on tyrosine-416, presumably reflecting suppression of c-Src activity, and examined the effects of short-term PP2 treatment of KM12L4A metastatic cells. At a concentration that effectively inhibited c-Src autophosphorylation in KM12L4A cells (Figure 4A

, upper panel), PP2 suppressed cell adhesion to fibronectin, but not to poly-L-lysine (Figure 4A, lower panel), while cell cycle distribution was not obviously affected (not shown). Thus, although PP2 is not a specific inhibitor of c-Src, or SFKs, it is able to effectively suppress c-Src activity without causing cell cycle withdrawal of KM12C cells. In addition, we showed that the more recently described selective Src inhibitor SU6656 (Blake *et al*, 2000), effectively suppressed both c-Src kinase activity and adhesion to fibronectin (Figure 4B). Suppression of KM12L4A cell–matrix adhesion by PP2 and SU6656 is consistent with a role for c-Src in the integrin-induced intracellular signal transduction cascade needed for the formation of cell–matrix adhesion structures in these colon cancer cells.

### Enforced expression of activated c-Src in the KM12C non-metastatic cells does not influence cell growth but stimulates assembly of integrin adhesions

**Figure 5:**
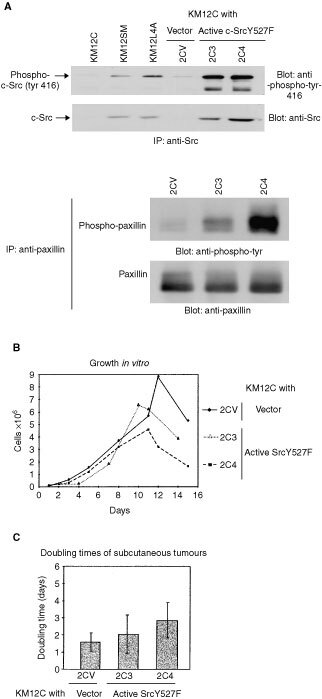
(**A**) c-Src expression and activity (monitored by auto-phosphorylation at tyrosine-416) in KM12C cell clones (2C3 and 2C4) stably expressing active c-SrcY527F, or vector control (2CV), was examined and compared with parental KM12C cells and their metastatic derivatives, KM12SM and KM12L4A. Paxillin phosphorylation in vector- and c-SrcY527F-expressing KM12C cells was also examined. (**B**) *In vitro* growth of KM12C cells stably expressing active c-SrcY527F (2C3 and 2C4; seeded at 1×10^5^ cells in 35 mm dishes) was compared with vector-transfected cells (2CV). (**C**) The doubling times of subcutaneous tumours formed after injection of 10^6^ KM12C cells transfected with either vector (2CV) or active c-SrcY527F (2C3 and 2C4) were determined.

**Figure 6:**
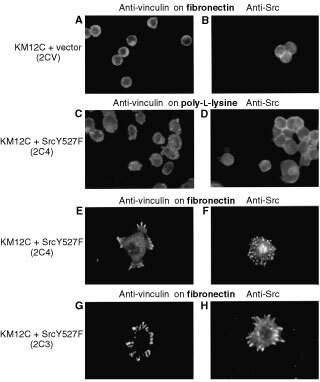
The effect of increasing cellular c-Src expression and activity on the formation of adhesion structures in KM12C cells expressing either vector (2CV; **A**, **B**) or active c-SrcY527F (2C3 or 2C4; **C**–**H**) were plated on to fibronectin (**A**, **B**, **E**, **F**, **G**, **H**) or poly-L-lysine (**C**, **D**), fixed and stained with anti-vinculin (**A**, **C**, **E** and **G**) or anti-Src (**B**, **D**, **F** and **H**) and examined by immunofluorescence. Bars are 100 μm.

To address whether elevated cellular c-Src activity was sufficient to induce formation of peripheral adhesion structures, we generated single cell clones of KM12C cells expressing an activated mutant of c-Src (SrcY527F; clones 2C3 and 2C4 are shown (Figure 5A

, upper panels). We confirmed that expression of activated c-Src in KM12C cells resulted in enhanced tyrosine phosphorylation of paxillin, although this was more pronounced in clone 2C4 (Figure 5B, lower panel). We found that KM12C non-metastatic cells (2CV) grew with similar kinetics to clones expressing c-SrcY527F (Figure 5B). Furthermore, the doubling times of vector- and c-SrcY527F-expressing KM12C cells grown *in vivo* as sub-cutaneous tumours were not significantly different in the mouse strain used and at the particular number of cells injected (Figure 5C). However, in contrast to the lack of growth stimulation, we found that KM12C cells expressing activated c-Src spread more readily and formed robust peripheral adhesions, as judged by anti-vinculin staining (Figure 6E

and G) or anti-Src staining (Figure 6F and H), after plating on fibronectin. This effect of c-SrcY527F expression was not evident when cells were plated on poly-L-lysine (Figure 6C and D), demonstrating integrin dependence. Vector-control transfected KM12C (2CV) cells spread poorly and remained relatively rounded (compare Figure 6A with E and G). These findings indicate that elevated expression of active c-Src in the non-metastatic KM12C cells is sufficient to confer an enhanced ability to spread on underlying matrix components by forming prominent integrin-dependent adhesions. Since this is also enhanced in the KM12L4A and KM12SM metastatic derivatives that express elevated c-Src (see Figure 3), it seems likely that this, rather than enhanced proliferation, may reflect the major contribution of elevated c-Src to metastatic potential in the Fidler model.

## DISCUSSION

Altered tyrosine phosphorylation of cellular proteins is associated with cell transformation, although exactly how individual tyrosine kinases contribute to aspects of the transformed phenotype in epithelial cancer cells, remains unclear. One particular oncoprotein that is frequently linked to colon cancer progression, and indeed to the progression of other epithelial cancers, is c-Src. Although the mode of increased c-Src expression and activity is not well understood, and may vary from cancer to cancer, it has been associated with different stages of colon tumour development, including metastasis (Bolen *et al*, 1987; Cartwright *et al*, 1989, 1990; Talamonti *et al*, 1993; Mao *et al*, 1997). Its precise role in the behavioural changes that colon cells undergo during malignant or metastatic transitions is also not defined.

We addressed whether elevated c-Src induced changes in cell growth or adhesion, both aspects of cell behaviour controlled by SFKs in fibroblasts that are frequently perturbed in cancer. We used the Fidler model of colorectal metastasis because it provides genetically related cell lines that differ in their intrinsic capacity to metastasise to the liver when injected into the spleen (Morikawa *et al*, 1988), and c-Src activity is elevated in the metastatic variants ((Mao *et al*, 1997); Figure 1A). Growth rate comparisons *in vitro*, under both anchorage-dependent and anchorage-independent conditions, and *in vivo* as sub-cutaneous tumours, did not reveal differences that correlated with elevated c-Src (Figure 2). Furthermore, when we expressed an activated mutant of c-Src (c-SrcY527F) in the non-metastatic cells, growth rates *in vitro* or *in vivo* were not increased (Figure 5), showing that elevating the intracellular tyrosine kinase activity of c-Src was not sufficient to promote growth. However, our data does not rule out that some endogenous c-Src activity might be required for the continued growth of colon cancer cells. It is possible, perhaps likely, that the expression and/or activity of c-Src has already been increased at an earlier stage of tumour progression (Cartwright *et al*, 1990). In this regard, there is evidence that inhibiting expression or activity of c-Src can suppress cancer cell growth (Park and Cartwright, 1995; Staley *et al*, 1997; Moasser *et al*, 1999). Taken together with our present findings, these data imply that while some c-Src activity might be required for proliferation of colon cancer cells, it is apparently not limiting in carcinoma cells from the Fidler model since growth is not enhanced by further elevation.

Examination of cell–matrix adhesion revealed that elevated c-Src correlated with, and resulted in, greater numbers of integrin-dependent, vinculin-containing peripheral structures in KM12 cells (Figures 3 and 6). The prominent peripheral adhesions that contain activated c-Src were dynamically regulated, compared with the static, more rudimentary peripheral adhesions in the parental non-metastatic cells, as judged by time-lapse imaging (not shown). Our recent results also suggest that elevated c-Src de-regulates E-cadherin-mediated adhesion between colon cancer cells, and that the integrin adhesion formation we describe here is required for this effect (Avizienyte *et al*, 2002). Thus, enhanced assembly of dynamically regulated cell–matrix adhesions may contribute to the behaviour of metastatic tumour cells in a number of ways. In this regard, c-Src co-operates with migratory growth factors, including epidermal growth factor (EGF), both to induce epithelial cell migration (Rodier *et al*, 1995), as well as to induce hepatocyte growth factor (HGF)- or EGF-induced invasion of colon cancer cells *in vitro* (Brunton *et al*, 1997; Empereur *et al*, 1997). Consequently, elevated c-Src activity might facilitate EGF- or HGF-induced biological effects by reducing the concentration of growth factor required to elicit an integrin-dependent migratory or invasive response, or render these processes independent of growth factors. However, expression of activated c-Src was not sufficient to convert non-metastatic cells into metastatic cells *in vivo* (not shown), indicating that while the adhesion changes induced by elevated c-Src might be contributory, these are not sufficient to elicit extravasation, attachment and growth at the ectopic liver site.

Although we have studied the role of elevated c-Src expression here in some detail in the genetically related cells of the Fidler model, our previous studies in another colon cancer progression model showed that c-Src itself is redistributed to integrin adhesions, whose formation accompanies acquisition of an invasive phenotype (Brunton *et al*, 1997). This together with the established role for c-Src in the mesenchymal transition of a rat bladder carcinoma cell line (Rodier *et al*, 1995), which is associated with loss of cell–cell and gain of cell–matrix adhesions, suggests that the role of c-Src in inducing matrix adhesion assembly is not restricted to cells from the Fidler model. Interestingly, in the rat bladder cancer model, c-Src is causally linked to the establishment of metastases (Boyer *et al*, 2002).

Our results imply that, at least for some advanced colon cancers, inhibitors of c-Src might be more likely to suppress further tumour spread than to inhibit tumour growth *per se*. Consequently, the conventional endpoints for pre-clinical and early phase clinical trials that monitor tumour shrinkage might not be the most appropriate for testing such agents.
